# The role of imaging in diagnosing an unusual manifestation of neurofibromatosis type 1: calvarial dysplasia

**DOI:** 10.1590/0100-3984.2016.0114

**Published:** 2018

**Authors:** Felipe Welter Langer, Daniel Mattos, Camila Piovesan Wiethan, Rafael Martins Scherer, Carlos Jesus Pereira Haygert

**Affiliations:** 1 Universidade Federal de Santa Maria (UFSM) - Radiologia e Diagnóstico por Imagem. Santa Maria, RS, Brazil

**Figure 1:**
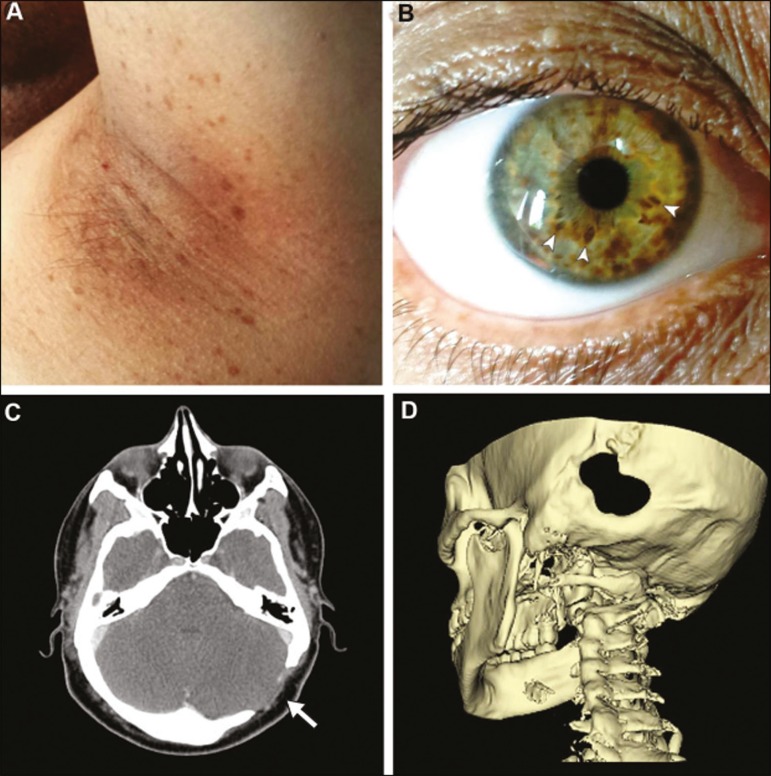
Clinical and CT findings. Bilateral axillary freckles (A) and iris hamartomas, also known as Lisch nodules (B, arrowheads) were detected, both meeting the criteria for a diagnosis of NF1. Axial and three-dimensional CT images (C and D, respectively) showing an occipital calvarial bone defect situated along the left lambdoid suture, measuring 3.1 cm at its largest diameter.

Dear Editor,

A 25-year-old man was referred to our institution for investigation of a one-year history of gradually developing nodules covering his skin. His medical history was unremarkable. On physical examination, multiple cutaneous nodules were noted, as were *café au lait* spots, bilateral Lisch nodules (Figure 1A), and axillary freckles (Figure 1B). The results of neurologic and fundoscopic examinations were unremarkable. The father of the patient, who was also examined, presented with similar cutaneous nodules. Those findings met the criteria for neurofibromatosis type 1 (NF1), which had previously gone undiagnosed. To determine the extent of the newly diagnosed NF1, computed tomography (CT) of the head was performed. The CT scan revealed an unsuspected left-sided discontinuity in the occipital bone along the left lambdoid suture (Figures 1C and 1D), measuring 3.1 × 2.7 cm. There were no signs of brain herniation through the bone aperture, and we detected no neurofibromas over the bone defect on the clinical examination or on the CT scan. Given the presence of NF1 and the absence of a history of neurologic surgery that could account for such a finding, a diagnosis of occipital calvarial dysplasia was established. Because the patient had no neurological symptoms, we opted for periodic clinical and imaging follow-up over surgical intervention. After one year of follow-up, the lesion remained unchanged and no neurologic symptoms had arisen.

NF1 is an autosomal dominant, multisystem disorder with extreme clinical heterogeneity^(1)^. The incidence of bony defects is higher in patients with NF1 than in the general population^(2)^, and studies in the recent radiology literature of Brazil have addressed the role of different imaging modalities in the evaluation of bone lesions^(3-6)^. However, although sphenoid wing dysplasia, pseudarthrosis of the tibia, and vertebral defects are hallmarks of NF1 and compose its standard diagnostic criteria, calvarial involvement in NF1 is uncommon^(1)^. Many patients with such defects are asymptomatic, although headache, visual symptoms, and concurrent bony skull defects can be present^(7)^. Therefore, a thorough clinical and imaging evaluation is called for when calvarial defects are detected.

Two hypotheses try to explain cranial vault defects in NF1^(8)^. One theory suggests that the calvarial lesions stem from an intrinsic bone development abnormality related to mutations within the NF1 gene itself. However, some authors postulate that NF1-related bone defects derive from increased exogenous pressure exerted by underlying neurofibromas, thereby leading to bone erosion and patency of cranial sutures. Because calvarial lesions have been observed in the presence and absence of adjacent tumors, it remains unclear whether these defects represent primary osseous dysplasia or pressure-induced responses to neurofibromas^(2,8)^.

Although the diagnosis of NF1 often relies on cardinal clinical findings, cross-sectional imaging studies can provide valuable information in sundry settings. Particularly for NF1 patients with skull defects, CT is essential for detecting and following up the lesions, given that progressive bone erosion occurs in more than half of all cases^(2)^ and such erosion can require calvarial reconstruction with bone grafts or titanium mesh^(1)^. However, progressive bone resorption can predispose to long-term implant instability, the best approach to NF1 calvarial defects therefore remaining undetermined^(2)^.
